# Improving prediction of secondary structure, local backbone angles, and solvent accessible surface area of proteins by iterative deep learning

**DOI:** 10.1038/srep11476

**Published:** 2015-06-22

**Authors:** Rhys Heffernan, Kuldip Paliwal, James Lyons, Abdollah Dehzangi, Alok Sharma, Jihua Wang, Abdul Sattar, Yuedong Yang, Yaoqi Zhou

**Affiliations:** 1 Signal Processing Laboratory, School of Engineering, Griffith University, Brisbane, Australia; 2 Institute for Integrated and Intelligent Systems, Griffith University, Brisbane, Australia; 3 School of Engineering and Physics, University of the South Pacific, Private Mail Bag, Laucala Campus, Suva, Fiji; 4 Shandong Provincial Key Laboratory of Functional Macromolecular Biophysics, Dezhou University, Dezhou, Shandong, China; 5 National ICT Australia (NICTA), Brisbane, Australia; 6 Institute for Glycomics and School of Information and Communication Technique, Griffith University, Parklands Dr. Southport, QLD 4222, Australia

## Abstract

Direct prediction of protein structure from sequence is a challenging problem. An effective approach is to break it up into independent sub-problems. These sub-problems such as prediction of protein secondary structure can then be solved independently. In a previous study, we found that an iterative use of predicted secondary structure and backbone torsion angles can further improve secondary structure and torsion angle prediction. In this study, we expand the iterative features to include solvent accessible surface area and backbone angles and dihedrals based on Cα atoms. By using a deep learning neural network in three iterations, we achieved 82% accuracy for secondary structure prediction, 0.76 for the correlation coefficient between predicted and actual solvent accessible surface area, 19° and 30° for mean absolute errors of backbone φ and ψ angles, respectively, and 8° and 32° for mean absolute errors of Cα-based θ and τ angles, respectively, for an independent test dataset of 1199 proteins. The accuracy of the method is slightly lower for 72 CASP 11 targets but much higher than those of model structures from current state-of-the-art techniques. This suggests the potentially beneficial use of these predicted properties for model assessment and ranking.

Three-dimensional structures for most proteins are determined by their one-dimensional sequences of amino acid residues. How to predict three-dimensional structures from one-dimensional sequences has been an unsolved problem for the last half century^1^. This problem is challenging because it demands an efficient technique to search in astronomically large conformational space and a highly accurate energy function to rank and guide the conformational search, both of which are not yet available^2^. As a result, it is necessary to divide the structure prediction problem into many smaller problems with the hope that solving smaller problems will ultimately lead to the solution of the big problem.

One of those smaller or sub-problems is the prediction of one-dimensional structural properties of proteins from their sequences. The most commonly predicted one-dimensional structural property of a protein is secondary structure. Secondary structure describes each amino residue in a number of discrete states^3^ for which three state description (helix, sheet and coil) is the most common. In recent years, there has been a slow but steady improvement of secondary structure prediction to above 81% when homologous sequences are not utilised for training (*ab initio* prediction)^4^,^5^. The steady improvement is due to a combination of improved machine-learning algorithms, improved features and larger training datasets. Other methods have also been developed to go beyond 81% by including homologous sequences in training^6^,^7^,^8^,^9^. Secondary structure directly predicted from sequence was shown more accurate than secondary structure of the models predicted by protein structure prediction techniques for template-free modelling targets in critical assessment of structure prediction (CASP 9)^4^.

Secondary structure, however, is a coarse-grained description of local backbone structure because ideal helical and strand conformations do not exist in protein structures and the boundary between coil states and helical/strand states is not well defined^10^. This leads to development of backbone torsion angle prediction (ϕ and ψ) in discontinuous^11^,^12^ and in real, continuous values^13^,^14^,^15^. More recently, a method for predicting angles based on Cα atoms (the angle between Cα_i−1_−Cα_i_−Cα_i+1_ (θ) and a dihedral angle rotated about the Cα_i_−Cα_i+1_ bond (τ)) was also developed^16^. These local structure descriptors are complementary with each other because torsion angles (ϕ and ψ), Cα−atom based angles (θ and τ), and secondary structure involve amino acid residues at different sequence separation: neighbouring residues for ϕ and ψ, 3–4 residues for θ and τ, and 4 for 3_10_ helix, 5 for α-helix, and an undefined number of residues for sheet residues.

Another important one-dimensional structure property is solvent Accessible Surface Area (ASA). ASA measures the level of exposure of an amino acid residue to solvent (water) in a protein. This is an important structural property as active sites of proteins are often located on their surfaces. Multistate prediction of earlier methods^17^,^18^,^19^ have been replaced by continuous real value prediction^14^,^20^,^21^,^22^,^23^.

One interesting observation is that predicted secondary structure is often utilized to predict other one-dimensional structural properties but rarely the other way around. Several studies, however, indicated that other predicted structural properties can be utilized to improve secondary structure prediction such as predicted torsion angles^4^,^13^ and predicted solvent accessible surface area^24^. In particular, we have shown that the accuracy of secondary structure and torsion angle prediction can be substantially improved by iteratively adding improved prediction of torsion angles and secondary structure^4^.

Artificial neural networks have been widely employed in predicting structural properties of proteins due to the availability of large datasets^25^. Deep neural networks^26^, referring to artificial neural networks with more than two hidden layers, have been explored in prediction of local and nonlocal structural properties of proteins^27^,^28^,^29^,^30^. For example, Qi *et al.*^29^ developed a unified multi-task, local-structure predictor of proteins using deep neural networks as a classifier. They trained a single neural network using sequential and evolutionary features to predict a number of protein properties including protein secondary structure and solvent accessibility. Spencer *et al.*^30^ developed an iterative deep neural network for protein secondary structure prediction. The method utilized one deep neural network to predict secondary structure by using physicochemical and evolutionary information in their first step and another deep neural network to predict their final secondary structure prediction based on predicted secondary structures in addition to the same input used in the first step. These methods achieved secondary structure prediction with accuracy that is slightly higher than 80%.

The goal of this paper is to develop an iterative method that predicts four different sets of structural properties: secondary structure, torsion angles, Cα−atom based angles and dihedral angles, and solvent accessible surface area. That is, both local and nonlocal structural information were utilized in iterations. At each iteration, a deep-learning neural network is employed to predict a structural property based on structural properties predicted in the previous iteration. We showed that all structural properties can be improved during the iteration process. The resulting method provides state-of-the-art, all-in-one accurate prediction of local structure and solvent accessible surface area. The method (named SPIDER2) is available as an on-line server at http://sparks-lab.org.

## Methods

This section describes the dataset employed and parametric details of the algorithm used as follows:

### Datasets

We employed the same training and independent test datasets developed for the prediction of Cα based angles (θ) and dihedral angles (τ)^16^. Briefly, a non-redundant (25% cutoff), high resolution (<2.0 Å) structures of 5789 proteins were obtained from the sequence culling server PISCES^31^ and followed by removing obsolete structures. We then randomly selected 4590 proteins as the training set (TR4590) and the remaining 1199 proteins as an independent test (TS1199). In addition, we downloaded the targets from critical assessment of structure prediction technique (CASP 11, 2014, http://www.predictioncenter.org/casp11/index.cgi). After removing the proteins with inconsistent sequences and the proteins with >30% sequence identities between each other and to the training and test sets (TR4590 and TS1199), we obtained a set of 72 proteins (CASP11) out of original 99 proteins. This set contains 17382 amino acid residues. A list of 72 proteins is provided in the Supplementary material.

### Deep neural-network learning

Here, we employed the same deep learning neural network as we have employed for prediction of Cα-based θ and τ angles prediction by SPIDER^16^. Briefly, the deep artificial Neural Network (ANN) consists of three hidden layers, each with 150 nodes. Input data was normalized to the range of 0 to 1. Weights for each layer were initialized in a greedy layer-wise manner, using stacked sparse auto-encoders which map the layer’s inputs back to themselves^32^ and refined using standard backward propagation. The learning rate for auto encoder stage was 0.05 and the number of epochs in auto encoder stage was 10. The learning rates for backward propagation were 1, 0.5, 0.2, and 0.05, respectively, with 30 epochs at each learning rate. In this study, we used the deep neural network MATLAB toolbox, implemented by Palm^33^. Linear activation function was used for the hidden layers of auto encoder training whereas sigmoid activation function was employed at the stage of back propagation. All these hyper parameters were obtained by a few initial studies of a single fold (90% for training and 10% for test), randomly selected from the training TR4590 dataset.

### Parallel multi-step iterative algorithm

**Figure 1:**
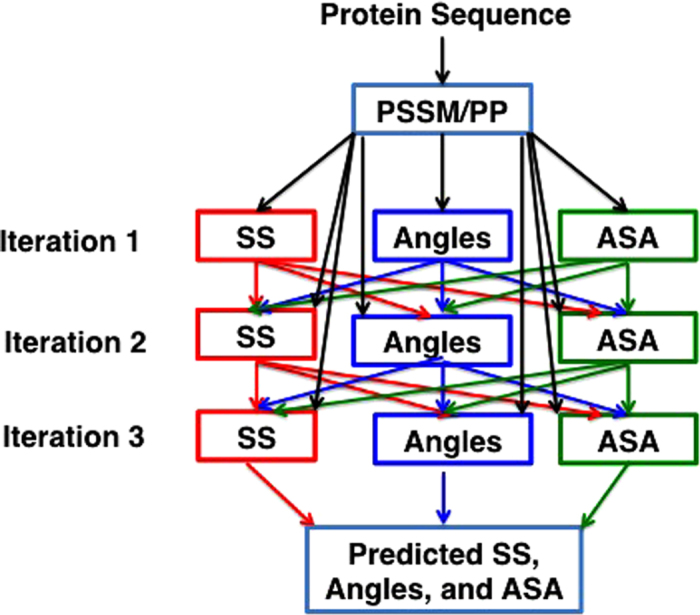
The general architecture of the parallel multi-step iterative algorithm. Black arrows indicate that position-specific scoring matrix (PSSM) and physical chemical properties (PP) are presented as input in every neural network predictor. There is no connection between each network.

Figure 1 shows the parallel, multi-step iterative algorithm for predicting secondary structure (SS), angles (backbone torsion angles, Cα-based angles) and ASA at the same time. In the first iteration, only seven representative physical chemical properties of amino acid residues^34^ and Position Specific Scoring Matrix (PSSM) from PSIBLAST^35^ were employed to predict SS, angles, and ASA, separately. The seven physicochemical properties (PP) of the amino acids employed are steric parameter (graph shape index), hydrophobicity, volume, polarizability, isoelectric point, helix probability, and sheet probability properties of the amino acids. PSSM was obtained by three iterations of searching against 90% non-redundant (NR90, ftp://toolkit.genzentrum.lmu.de/pub/HH-suite/databases/nr90.tar.gz) protein data bank with a cut off value (so called E-value) set to 0.001. PSSM represents the substitution probability of a given amino acid based on its position in the protein sequence with all 20 amino acids.

In the second iteration, PSSM/PP plus predicted SS, angles, and ASA from the first iteration were employed to predict SS, angles, and ASA, separately. Additional iterations can be followed by using SS, angles, and ASA from the previous iteration in addition to PSSM and PP. We found three iterations are sufficient for achieving the best predictive power. Thus, each iteration has three separate predictors. Each predictor utilizes one stacked auto-encoder deep neural network as described above.

### Input

We employed a window size of 17 amino acids (8 amino acids at each side of the target amino acid). For the residues on terminal ends of a protein sequence, we simply repeat the residue type of the first (or last) residue to fill the window. This led to a total of 459 input features (17 × (20 PSSM + 7PP)) for a given amino acid residue in the first iteration. This window size was optimized by 10-fold cross validation. The dependence on window size is small. For example, the accuracy of secondary structure prediction for the first iteration is 80.4–80.5% for the window size of 13, 15, 17, 19, and 21.

### Output

For output nodes, the SS predictor has three output nodes representing helix, strand, and coil, respectively; the ASA predictor has only one output node, and the angle predictor has eight output nodes representing sin(θ), cos(θ), sin(τ), cos(τ), sin(ϕ), cos(ϕ), sin(ψ), and cos(ψ), respectively. Sine and cosine were employed to remove the effect of angle periodicity. Predicted sine and cosine values are converted back to angles by using the equation *α* = tan^−1^[sin(*α*)/cos(*α*)]. In the second iteration, the number of inputs for each predictor is 663 (=17 × (20 PSSM + 7 PP + 3 SS + 1 ASA + 8 Angles)). Only sin(θ), cos(θ), sin(τ), cos(τ), sin(ϕ), cos(ϕ), sin(ψ), and cos(ψ) are utilised in the input for angles. The same number of inputs was employed for additional iterations.

### Ten-fold cross validation and independent test

The method was first examined using ten-fold cross validation where TR4590 was randomly divided into 10 folds. Nine folds were used in turn for training and the remaining one for test until all 10 folds were tested. In addition, we tested our method for the independent test sets TS1199 and CASP11 by using TR4590 as the training set.

### Performance measure

For secondary structure, we use the fraction of correctly predicted secondary structure elements for accuracy measurement (Q3)^3^. The accuracy of predicted angles was measured by a Mean Absolute Error (MAE), the average absolute difference between predicted and experimentally determined angles. The periodicity of an angle was taken care of by utilizing the smaller value of the absolute difference 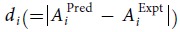 and 360 − d_*i*_ for average. For ASA, we report both MAE and the Pearson correlation coefficient between predicted and actual ASA.

## Results

**Figure 2:**
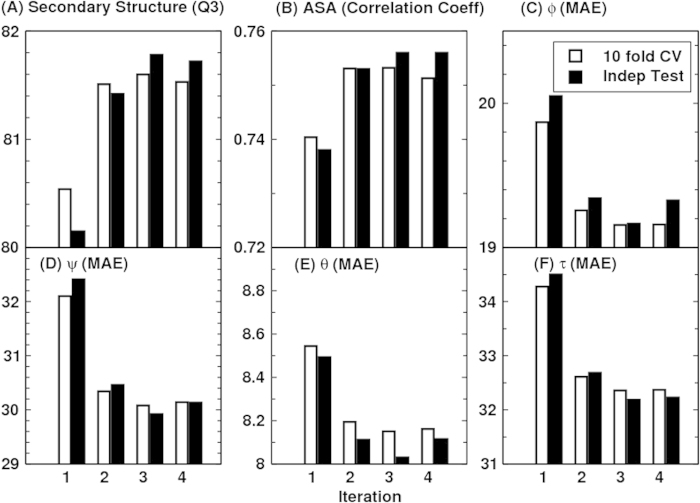
The accuracy of secondary structure (Q3), ASA (correlation coefficient), ϕ (Mean absolute error, MAE), ψ (MAE), θ (MAE) and τ (MAE) at four different iterations. Open and filled bars denote results from 10 fold cross validation and independent test, respectively.

The overall accuracy for all six structural properties (secondary structure, ASA, ϕ, ψ, θ, and τ) as a function of iterations is shown in Fig. 2. The improvement is clear at the second iteration and converged at the third iteration, regardless if it is 10 fold cross validation or independent test. Thus, we stopped the iteration at the third iteration. Three iterations led to about 1% improvement in Q3. In Table 1, we monitored the accuracy of each amino acid residue for secondary structure prediction. We found that for 17 of 20 amino acids, the accuracy improves in all three iterations. This confirms the robustness of improvement by iterations.

In addition to improvement in secondary structure prediction, there is a 2% improvement in ASA correlation coefficient, 1°, 2°, 0.5° and 2° improvement in ϕ,ψ, θ, and τ, respectively. Improvement in angles is the most significant, representing 5%–6%, relative improvement. At the third iteration, Q3 for the secondary structure is 81.6% for 10 fold cross validation and 81.8% for the independent test. The correlation coefficient between predicted and measured ASA is 0.751 for 10 fold cross validation and 0.756 for independent test. This is the correlation coefficient for un-normalized ASA. For normalized ASA (rASA), the correlation coefficient is slightly lower (0.731 for the independent test set). The mean absolute error for rASA is 0.145. The mean absolute errors of the angles for 10 fold cross validation (or independent test) are 19.2° (19.2°) for ϕ, 30.1° (29.9°) for ψ, 8.15° (8.03°) for θ, 32.4° (32.2°) for τ. Similar accuracy between 10 fold cross validation and independent test indicates the robustness of the method being developed.

It is of interest to know if this improvement in angle MAE also translates into improvement in large angle errors. Reducing large angle errors is essential for sampling in the correct conformational space when used as restraints. Because both ϕ and ψ have two peaks in their distributions, they can be divided into two states associated with the two peaks. Here we define [0° to 150°] and the rest angle range [(150° to 180°) and (−180° to 0°)] for two states in ϕ, and [−100° to 60°] and the rest angle range [(−180° to −100°) and (60° to 180°)] for two states in ψ. We found that for the independent test set, the two-state accuracy for ϕ only increases marginally from 96.4%, 96.5% to 96.6% from the first to the third iteration. The two-state accuracy for ψ increases by a significant 1% from 85.3% , 86.4% to 86.8%. This significant increase confirms the usefulness of iterative learning. By comparison, SPINE-X^36^ was trained for two-state prediction and achieved two state accuracy of 96.4% for ϕ and 85.6% for ψ.

**Figure 3:**
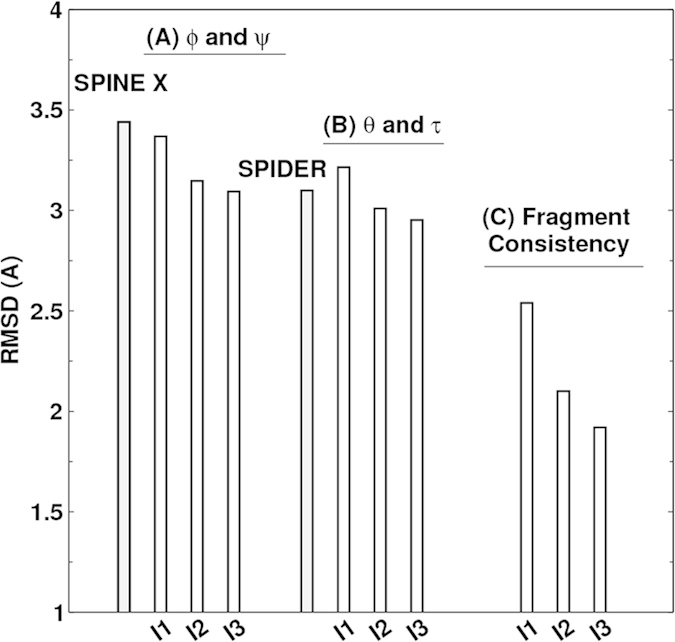
The improvement of fragment structures of 15 residues for the TS1199 dataset : (**A**) RMSD between the native fragments and the fragments generated from predicted ϕ and ψ for three iterations I1, I2, and I3 and the result from SPINE X (in grey bar). (**B**) RMSD between the native fragments and the fragments generated from predicted θ and τ for three iterations I1, I2, and I3. (**C**) The consistency between fragments from predicted ϕ and ψ and fragments from predicted θ and τ for three iterations I1, I2, and I3.

Once ϕ and ψ or θ and τ are known protein backbone structure can be constructed. Fragment structures of a length L are derived from predicted angles with a sliding window (1 to L, 2 to L + 1, 3 to L + 2, and etc.). For L = 15, a total of 229,681 fragments are constructed. For ϕ/ψ derived fragments, each fragment structure was built by standard bond lengths and angles and ω = 180°. For θ/τ derived fragments, each fragment structure was built by the standard Cα-Cα distance of 3.8 Å. The accuracy of a fragment structure can be measured by root-mean-squared distance (RMSD) from the corresponding native fragment. The accuracy of fragment structures either from ϕ and ψ (Fig. 3A) or from θ and τ (Fig. 3C) improves during iterations (from 3.37 to 3.09 Å for ϕ/ψ derived fragments and from 3.22 to 2.95 Å for θ/τ derived fragments. Perhaps, not surprisingly, the consistency between ϕ/ψ and θ/τ derived fragments has the largest improvement during iterations (from to 2.54 Å to 1.92 Å). Results for other sizes of fragments follow similar trend. This further confirms the power of iterative learning.

Our method is further applied to the most recent CASP targets (CASP11, 2014). It achieves 80.8% in secondary structure, 0.74 for correlation coefficient between measured and predicted ASA, 19.7° MAE for ϕ, 30.3° for ψ, 8.2° for θ, 32.6° for τ. The prediction accuracy for most structural properties is reduced somewhat from the independent test set to CASP 11 set. This type of reduction for CASP sets was observed previously^4^. This is in part due to a smaller number of targets (72 proteins) and in part because CASP targets were a carefully selected set for challenging structure prediction techniques.

Tables 2 and 3 compare our method with several techniques for secondary structure (PSIPRED3.3^37^, SCORPION^38^, SPINE-X^4^), ASA (SPINE-X^4^), backbone torsion angles (SPINE-X^4^) and backbone Cα angles and dihedral angles (SPIDER^16^) for TS1199 and CASP11 test datasets. We noted that TS1199 is not necessarily independent test set for other methods. In fact we found that the majority of TS1199 (all but 66 proteins) are contained in the training set for SCORPION. The accuracy for secondary structure predicted by our method is more accurate than that predicted by PSIPRED and SPINE-X and is comparable to SCORPION for the full TS1199 dataset. However, for the 66 proteins not trained by SCORPION, it achieves an accuracy of 82.4%, compared to 83.3% by our method. For ASA prediction, our technique continues to make an improvement over SPINE-X despite its high accuracy. The best improvement over previous methods is angle prediction. For example, there is almost 4° degree improvement (>10% in relative improvement) over SPINE-X in ψ prediction. It is important to know the statistical significance of the difference among different methods. The p-values for the pair t-test in secondary structure of this work to SCORPION, PSIPRED, and SPINE X are 0.036, 0.00006, and 0.00009, respectively. That is, the improvement from this work over previous methods is statistically significant (<0.05).

**Figure 4:**
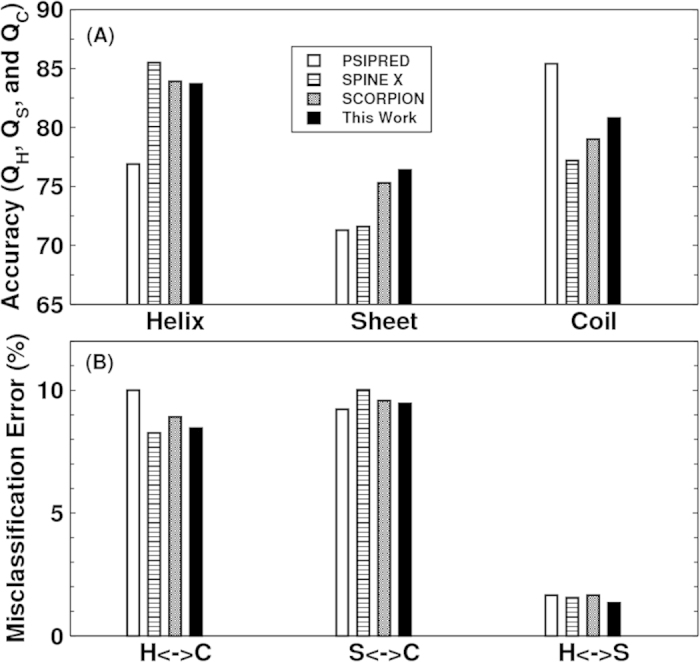
(**A**) The accuracy of helical, sheet and coil residues predicted by PSIPRED, SPINE X, SCORPION and the present study for the CASP 11 dataset. (**B**) The misclassification errors between helix and coil, between sheet and coil and between helix and sheet for the four methods as labelled for the CASP11 dataset.

In Fig. 4A we compare the accuracy of secondary structure prediction for helix, coil and sheet given by four methods for the CASP11 dataset (PSIPRED, SPINE X, SCORPION and present study). Our method provides the highest accuracy for sheet (76.4%) but lower accuracy in helical prediction (83.7%) than SPINE X (85.5%) and lower accuracy in coil prediction (80.8%) than PSIPRED (85.4%). PSIPRED is significantly more accurate in coil prediction because it over-predicts coil residues^4^.

Figure 4B further compares misclassification errors associated with different methods. This confirms that our method gives lower error in misclassification between helix and sheet. It gives a comparable error to SCORPION between sheet and coil and to SPINE X between helix and coil.

**Figure 5:**
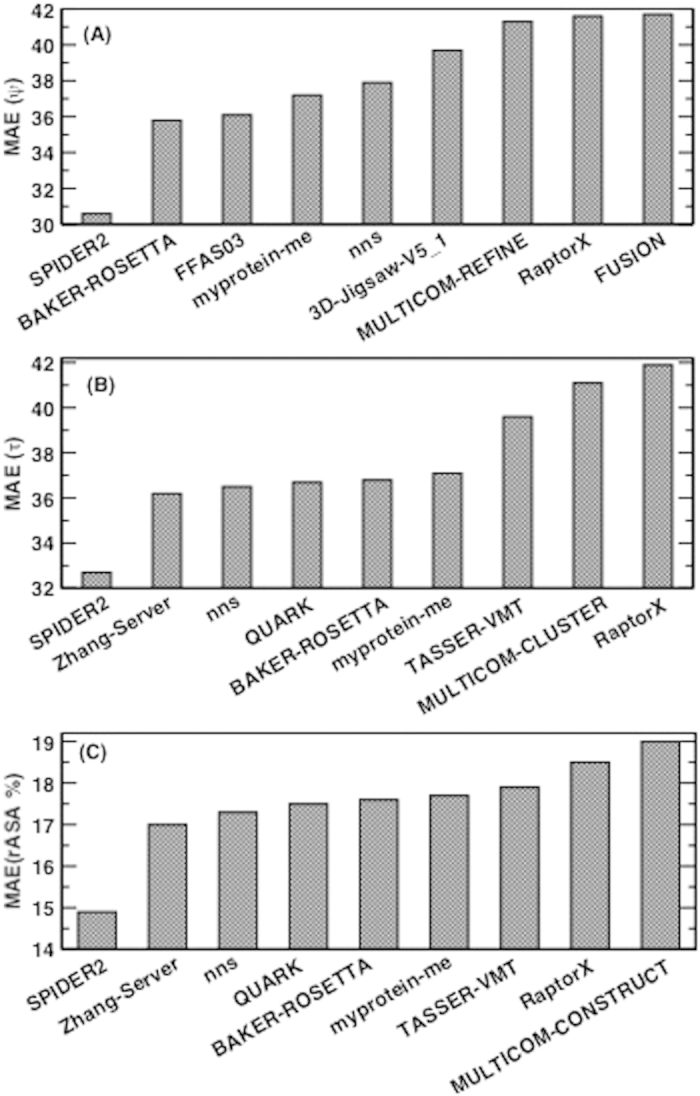
(**A**) The mean absolute error (MAE) of predicted ψ for the CASP 11 dataset compared to best MAEs of ψ angles in the models from eight most accurate methods in CASP 11. (**B**) as in (**A**) but for the MAE of Cα based τ angles. (**C**) as in (**A**) but for the MAE of relative assessable surface area (rASA).

It is of interest to know how the predicted values are compared to those of models in CASP 11. Methods compared are Zhang-server^39^, BAKER-ROSETTA^40^, FFAS^41^, myprotein-me (http://myprotein.me), nns^42^, 3D-Jigsaw^43^, RaptorX^44^, Quark^45^, TASSER^46^, and Fusion/MULTICOM^47^. Figures 5A,B shows that the MAE of predicted ψ and τ angles are 14% and 10% smaller than the lowest MAEs from BAKER-ROSETTA^40^ and Zhang Server^39^, respectively. Figure 5C further shows that predicted relative ASA values are also 12% better than those of model structures.

**Figure 6:**
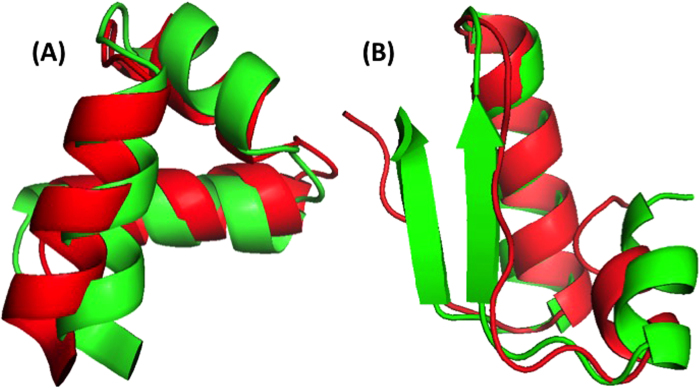
The structure (Red) constructed directly from ϕ/ψ angles compared to native structure (Green). (**A**) Residues 174 to 213 from PDB 1l3l chain A and (**B**) Residues 77 to 116 from PDB 1jq5 chain A.

The significant improvement in fragment structures revealed in Fig. 3 leads to an interesting question: can predicted angles be directly employed in building accurate protein structures? The direct answer to this question is no because accumulation of errors in angles can lead to large deviation in three-dimensional structures. On the other hand, there is a small chance for cancellation of errors. The test dataset (1199 proteins) has 183924 40-mer fragments. The percentages of 40-mer fragments with a RMSD below 2.5Å by ϕ/ψ and θ/τ are 1.4% and 1.6%, respectively. In Fig. 6A, a 40-residue fragment of a three helical bundle constructed based on predicted ϕ/ψ angles (Residues 174 to 213 from PDB 1l3l chain A) is only 2.2Å RMSD from the native structure. Figure 6B shows an example of a mixed helix/strand fragment of 40 residues (Residues 77 to 116 from PDB 1jq5 chain A). The RMSD between predicted and native structure is 2.4 Å. The two constructed structures show that helical structures are more accurately reproduced than strands. What is most encouraging in Fig. 6A,B is well reproduced loop and turn regions that permitted accurate reproduction of fragments.

## Discussion

This paper developed an integrated sequence-based prediction of one-dimensional structural properties of proteins by iterative learning in a parallel scheme. The structural properties include local backbone structures represented by secondary structure, backbone torsion angle, and backbone Cα angles and dihedral angles. These three backbone representations are complementary to each other: backbone torsion angles are single residue properties, backbone Cα angles and dihedral angles involve three and four residues, respectively, and secondary structures involve three or more residues in sequence-position separation. In addition, the method predicts a non-local property: solvent accessibility. We have shown that the input of these predicted structural properties can improve the accuracy of these structural properties iteratively (within three iterations).

The method provides current state-of-the-art prediction accuracy for various structural properties. For secondary structure prediction, its accuracy reaches nearly 82% for the large test set of 1199 proteins. For solvent accessible surface area, the correlation coefficient between predicted and actual ASA values is 0.76. For angles, MAEs for ϕ, ψ, θ, and τ are 19.2, 29.9, 8.0 and 32.2 degrees, respectively. Application to a small but more challenging dataset of CASP 11 targets leads to only slightly lower accuracy. All these accuracies above are the best reported accuracies for test sets. Such an integrated collection of various predicted structural properties in one server makes it convenient for their use for other applications.

One interesting question is that whether or not improvement from iterations is due to addition of predicted secondary structures or other predicted structural information. Spencer *et al.*^30^ showed that adding predicted secondary structures alone is sufficient to further improve secondary structure prediction. We performed independent tests by removing other non-secondary-structural features and achieved Q_3_ = 81.2% in the second iteration and Q_3_ = 81.2% in the third iteration, compared to 81.4% at the second iteration and 81.8% at the third iteration with non-secondary-structural features. This indicates that adding predicted secondary structures alone contributes a large portion of the improvement whereas other features lead to additional improvement.

One obvious application is protein structure prediction. Previously, we have shown that predicted secondary structures are more accurate than the models predicted by various current state-of-the-art techniques^4^. Here we demonstrate that the same is true for backbone angles and solvent accessibility (Fig. 5). Indeed, employing predicted torsion angles as restraints doubled the success rate in *ab initio* structure prediction, compared to using predicted secondary structures^36^. This success was because continuous angles can capture not only non-ideal conformations of helical/strand residues but also essential structural information of coil residues. Such structural information is essential for correct folding of a three-dimensional structure as demonstrated in Fig. 6. Predicted angles and solvent accessibility were also found useful in template-based structure prediction^48^.

## Additional Information

**How to cite this article**: Heffernan, R. *et al.* Improving prediction of secondary structure, local backbone angles, and solvent accessible surface area of proteins by iterative deep learning. *Sci. Rep.* **5**, 11476; doi: 10.1038/srep11476 (2015).

## Supplementary Material

Supplementary Information

## Figures and Tables

**Table 1:** The accuracy of predicted secondary structure for each amino acid residues for TS1199 for 4 iterations.

			Secondary Structure Iteration			
Amino acids	Abundance	Frequency	1	2	3	4
A	21477	8.27	82.3	83.4	83.8	83.6
C	3557	1.37	74.4	75.3	76.3	76.6
D	15271	5.88	80.8	81.9	82.4	82.1
E	17413	6.71	81.5	83.0	83.5	83.3
F	10457	4.03	78.3	79.3	79.9	80.2
G	18723	7.21	80.6	81.8	82.1	82.0
H	5942	2.29	77.3	78.1	78.1	78.8
I	14577	5.61	82.2	83.4	84.0	83.8
K	15216	5.86	79.7	81.2	81.7	81.4
L	23835	9.18	81.7	83.3	83.6	83.4
M	5615	2.16	80.2	81.5	81.8	82.1
N	11306	4.35	79.8	80.8	80.8	80.9
P	11860	4.57	81.1	82.6	83.2	82.8
Q	9927	3.82	79.7	81.7	81.0	81.3
R	13307	5.12	79.6	81.0	81.2	81.5
S	15363	5.92	77.3	78.6	79.0	78.7
T	14445	5.56	77.8	78.9	79.0	79.1
V	18270	7.04	82.0	83.4	83.6	83.5
W	3828	1.47	76.7	78.3	79.4	78.8
Y	9273	3.57	76.8	78.4	79.0	79.0
Overall	259662	100.0	80.2	81.4	81.8	81.7

**Table 2:** Accuracy comparison between our technique and several techniques for secondary structure, ASA and angle prediction for the independent test set (TS1199).

Method	PSIPRED	SPINE-X	SCORPION	SPIDER	This Work
SS (Q3)	79.7%(80.8%^a^)	81.0%(80.6%^a^)	82.0%(82.4%^a^)		81.8%(83.3%^a^)
ASA (CC)	–	0.74	–	–	0.76
MAE: ϕ(°)	–	20.2	–	–	19.2
MAE: ψ(°)	–	33.7	–	–	29.9
MAE: θ(°)	–	–	–	8.6	8.0
MAE: τ(°)	–	–	–	33.6	32.2

^a^66 proteins of TS1199 that are not in the training set for SCORPION.

**Table 3:** Accuracy comparison between our technique and several techniques for secondary structure, ASA and angle prediction for the independent CASP11 set.

Method	PSIPRED	SPINE-X	SCORPION	SPIDER	This Work
SS (Q3)	78.8%	78.8%	79.9%		80.8%
ASA (CC)	–	0.72	–	–	0.74
MAE: ϕ(°)	–	20.7	–	–	19.7
MAE: ψ(°)	–	34.6	–	–	30.3
MAE: θ(°)	–	–	–	8.7	8.2
MAE: τ(°)	–	–	–	34.1	32.6
