# Supplementary material for: Improving prediction of secondary structure, local backbone angles, and solvent accessible surface area of proteins by iterative deep learning
Source: Sci Rep. 2015 Jun 22;5:11476. doi: 10.1038/srep11476 (PMC4476419; doi:10.1038/srep11476)
Supplement: Supplementary Information [file srep11476-s1.pdf]

Supplementary material for "Improving prediction of secondary structure, local backbone angles, and solvent accessible surface area of proteins by iterative deep learning." *Rhys Heffernan, [a] Kuldip Paliwal, [a] James Lyons,[a] Abdollah Dehzangi, [a,b] Alok Sharma,[b,c] Jihua Wang, [d] Abdul Sattar,[b,e] Yuedong Yang*[f] and Yaoqi Zhou*[d,f],*[a] Signal Processing Laboratory, School of Engineering, Griffith University, Brisbane, Australia,[b]Institute for Integrated and Intelligent Systems, Griffith University, Brisbane, Australia ,[c]School of Engineering and Physics, University of the South Pacific, Private Mail Bag, Laucala Campus, Suva, Fiji ,[d] Shandong Provincial Key Laboratory of Functional Macromolecular Biophysics, Dezhou University, Dezhou, Shandong, China ,[e]National ICT Australia (NICTA), Brisbane, Australia ,[f] Institute for Glycomics and School of Information and Communication Technique, Griffith University, Parklands Dr. Southport, QLD 4222, Australia.,E-mail: yaoqi.zhou@griffith.edu.au or yuedong.yang@griffith.edu.au.

List of 72 CASP 11 targets

T0759, T0760, T0761, T0762, T0763, T0764, T0765, T0766, T0767, T0768, T0769, T0770, T0771, T0772, T0774, T0776, T0777, T0780, T0781, T0782, T0783, T0785, T0786, T0789, T0791, T0792, T0794, T0796, T0800, T0801, T0803, T0805, T0806, T0807, T0808, T0810, T0812, T0813, T0814, T0815, T0816, T0817, T0818, T0819, T0820, T0821, T0822, T0823, T0824, T0827, T0829, T0830, T0831, T0832, T0833, T0834, T0835, T0836, T0837, T0838, T0843, T0845, T0847, T0848, T0849, T0851, T0852, T0853, T0854, T0855, T0856, T0857.
